# Extent of Surgery and the Prognosis of Unilateral Papillary Thyroid Microcarcinoma

**DOI:** 10.3389/fendo.2021.655608

**Published:** 2021-06-16

**Authors:** Hengqiang Zhao, Le Cui

**Affiliations:** Department of Breast and Thyroid Surgery, Renmin Hospital of Wuhan University, Wuhan, China

**Keywords:** papillary thyroid microcarcinoma, total thyroidectomy, lobectomy, prognosis, propensity score matching

## Abstract

It remains controversial whether patients with papillary thyroid microcarcinoma (PTMC) benefit from total thyroidectomy (TT) or thyroid lobectomy (TL). We aimed to investigate the impact of extent of surgery on the prognosis of patients with unilateral PTMC. Patients were obtained from the Surveillance, Epidemiology, and End Results database from 2004 to 2015. Cancer-specific survival (CSS) and overall survival (OS) were evaluated by Cox regression and Kaplan–Meier curves with propensity score matching. Of 31167 PTMC patients enrolled, 22.2% and 77.8% of which underwent TL and TT, respectively. Patients with TT were more likely to be younger, females, present tumors of multifocality, extrathyroidal extension, cervical lymph node metastasis (CLNM), distant metastasis, and receive radioactive iodine (RAI) compared with those receiving TL. The multivariate Cox regression model showed that TT was not associated with an improved CSS and OS compared with TL with hazard ratio (HR) and 95% confidence interval (CI) of 0.53 (0.25-1.12) and 0.86 (0.72-1.04), respectively. In addition, the Kaplan–Meier curves further confirmed the similar survival between TL and TT after propensity score matching. The subgroup analysis showed that TT was associated with better CSS for patients < 55 years, those with tumors of gross extrathyroidal extension, CLNM (N1b), and cases not receiving RAI with HR 95% CI of 0.13 (0.02-0.81), 0.12 (0.02-0.66), 0.11 (0.02-0.64) and 0.36 (0.13-0.90), respectively. TT predicted a trend of better OS for patients with N1b and distant metastasis after adjustment. In addition, TT was associated with better CSS than TL for patients with risk factors like N1b combined with gross extrathyroidal extension, and/or multifocality after matching. In conclusion, TL may be enough for low-risk PTMC patients. TT may improve the prognosis of unilateral PTMC patients with 2 or more risk clinicopathologic factors like CLNM, multifocality, extrathyroidal extension and a younger age compared with TL.

## Introduction

Papillary thyroid microcarcinoma (PTMC) is defined as a papillary thyroid carcinoma (PTC) ≤ 1.0 cm in diameter, which has been increasingly detected in recent decades across the world with the popularity of ultrasound and fine-needle aspiration cytology (1, 2). PTMC is generally an indolent disease with excellent prognosis (3). Recently, active surveillance has been recommended as an alternative approach for low-risk PTMC according to the American Thyroid Association (ATA) guidelines (4).

Thyroid lobectomy (TL) alone was sufficient for unifocal and intrathyroidal PTMC in the absence of clinically detectable cervical nodal metastasis (4). TL may be appropriate for PTMC patients when no evidence of extrathyroidal disease was found (5). However, The rate of pathological cervical lymph node metastasis (CLNM) was 48.0% for PTC (6), and 42.4% for PTMC when prophylactic central lymph node dissection was performed (7), which does pose a risk for local recurrence (8). Postoperative local lymph node recurrence was associated with reoperations and the consequently excess morbidity from reoperations (9).

Besides TL, total thyroidectomy (TT) is commonly performed on unilateral PTMC patients. TT was associated with more complications like hypocalcemia, recurrent laryngeal nerve injury, and high-dose hormone replacement throughout one’s life. However, it remains unclear whether patients with unilateral PTMC benefit from TL or TT (10). We expect that PTMC patients with risk clinicopathologic features may benefit from more aggressive surgical treatment. However, it remains unclear due to the excellent prognosis of PTMC and limited qualified cases. In this study, we aim to compared the prognosis between patients receiving TT and TL with a large sample size.

## Patients and Methods

### Ethics Statement

The patients were enrolled from the Surveillance, Epidemiology, and End Results (SEER) program (https://seer.cancer.gov/) from 2004 to 2015. This study was deemed exempt by the institutional review board approval for the deidentified patient information.

### Study Population

Medical records were drawn using the International Classification of Diseases for Oncology code site C73.9. Histotype of PTC with values of 8050 (papillary carcinoma), 8260 (papillary adenocarcinoma), 8340 (papillary carcinoma, follicular variant), and 8341 (papillary microcarcinoma) were included. Values of 8050, 8260 and 8341 were classified as PTC, and 8340 for follicular variant PTC (FVPTC). The demographic, clinicopathologic characteristics, and treatment along with survival data were recorded. Race was categorized into white, black, and other (American Indian/AK Native, Asian/Pacific Islander). Extrathyroidal extension was divided into minimal extension and gross extension. Cervical lymph node metastasis was determined by derived AJCC N stage, 6^th^ ed (2004+), which includes N0 (without nodal metastasis) and N1 (N1a, N1b, and N1). N1a means nodal metastasis to level VI (pretracheal, paratracheal, and prelaryngeal/Delphian lymph nodes). N1b represents nodal metastasis to unilateral, bilateral, or contralateral cervical or cervical or superior mediastinal lymph nodes. N1 (NOS) means regional nodal metastasis. Patients with multiple primary tumors, tumors in both sides of thyroid lobes, non-positive histology, age < 18 years, unknown or indefinite data of interest were excluded. Only patients with unilateral PTMC were included.

### Statistical Analysis

Age and year of diagnosis were expressed as median (upper and lower quartile) for its skewed distribution and analyzed with Mann-Whitney *U* test. Category variables were presented as percentage and analyzed using chi-square test. The cancer specific survival (CSS) and overall survival (OS) were estimated by the Kaplan–Meier curves and compared by log-rank tests. The Cox proportional hazard model was established to estimate risk factors for CSS and OS with hazard ratio (HR) and a 95% confidence interval (CI). Propensity score matching (PSM) was performed using R software (ver. 3.3.3, http://www.r-project.org/) of package ‘MatchIt’. One-to-one matching with a caliper of 0.1 was used to balance demographic, pathologic and treatment covariates between TL and TT (11). The matched variables included age, year of diagnosis, sex (male *vs.* female), multifocality (solitary and multiple nodules), extrathyroidal extension (no *vs.* yes), cervical lymph node metastasis (no *vs.* yes), distant metastasis (no *vs.* yes), and radioactive iodine (RAI). Subgroup analyses stratified by clinicopathologic characteristics were performed (12). All statistical differences were set at a two-sided *p* value < 0.05. The other data were analyzed by Stata software (Stata/MP ver. 14.2, StataCorp., College Station, TX), and GraphPad Prism (ver 7.0, GraphPad Software, Inc).

## Results

### Patient Characteristics

The flow chart of selection was shown in **Supplementary Figure 1**. Finally, a total of 31167 patients with unilateral PTMC were enrolled, including 6929 (22.2%) undergoing TL and 24238 (77.8%) undergoing TT (**Table 1**). The following characteristics of patients were more likely to present with TT compared with TL: younger age, later year of diagnosis, female sex, tumors of multifocality, extrathyroidal extension (minimal and gross extension), CLNM (N1a, N1b and N1), distant metastasis, and treatment with RAI (**Table 1**).

**Table 1:** The clinicopathologic features of PTMC patients treated with TL *versus* TT.

Variables	TL (*n* = 6929) *n* (%)	TT (*n* = 24238) *n* (%)	*P-*value
Age (year)	51 (41-61)	49 (40-58)	<0.001
<55	4179 (60.3)	15762 (65.0)	<0.001
≥55	2750 (39.7)	8476 (35.0)	
Year of diagnosis	2010 (2007-2013)	2011 (2008-2013)	<0.001
Sex			
Male	1322 (19.1)	4029 (16.6)	<0.001
Female	5607 (80.9)	20209 (83.4)	
Race			
White	5753 (83.0)	20316 (83.8)	0.221
Black	463 (6.7)	1591 (6.6)	
Other	713 (10.3)	2331 (9.6)	
Multifocality			
No	5705 (82.3)	14355 (59.2)	<0.001
Yes	1224 (17.7)	9883 (40.8)	
Extrathyroidal extension			
No	6805 (98.2)	22527 (92.9)	<0.001
Minimal extension	56 (0.8)	866 (3.6)	
Gross extension	68 (1.0)	845 (3.5)	
CLNM			
No	6814 (98.3)	20758 (85.6)	<0.001
N1a	70 (1.0)	1917 (7.9)	
N1b	22 (0.3)	1212 (5.0)	
N1 (NOS)	23 (0.3)	351 (1.4)	
Distant metastasis			
No	6923 (99.9)	24180 (99.8)	0.013
Yes	6 (0.1)	58 (0.2)	
Histotype			
PTC	4842 (69.9)	17171 (70.8)	0.121
FVPTC	2087 (30.1)	7067 (29.2)	
RAI			
No	6582 (95.0)	15931 (65.7)	<0.001
Yes	347 (5.0)	8307 (34.3)	
Chemotherapy			
No	6925 (99.9)	24215 (99.9)	0.354
Yes	4 (0.1)	23 (0.1)	

Age and year of diagnosis were expressed as median with interquartile, other variables were expressed as n (%). PTMC, papillary thyroid microcarcinoma; TL, Thyroid lobectomy; TT, total thyroidectomy; CLNM, cervical lymph node metastasis; PTC, papillary thyroid carcinoma; FVPTC, follicular variant papillary thyroid carcinoma; RAI, radioactive iodine.

### Predictors for CSS and OS of Patients With PTMC

Results showed that increasing age, gross extrathyroidal extension, N1a, N1b, distant metastasis, treatment with RAI, and chemotherapy were associated with compromised CSS in PTMC patients with HR (95% CI) of 1.12 (1.09-1.14), 2.60 (1.22-5.56), 4.69 (1.92-11.5), 10.11 (4.64-22.03), 24.54 (11.17-53.89), 2.56 (1.25-5.25) and 14.10 (3.03-58.09) compared with the corresponding counterparts in the multivariate Cox regression (**Table 2**). In addition, increasing age, male sex, black race, tumors of gross extrathyroidal extension, N1a, N1b, N1 (NOS), distant metastasis, and treatment with chemotherapy were associated with compromised OS in PTMC patients with HR (95% CI) of 1.10 (1.09-1.10), 1.68 (1.41-2.00), 1.99 (1.54-2.56), 1.67 (1.15-2.41), 1.29 (0.85-1.94), 2.40 (1.68-3.43), 6.19 (3.54-10.82), and 3.64 (1.12-11.80) relative to the corresponding groups after adjustment (**Table 2**).

**Table 2:** The predictors for CSS and OS of PTMC patients by multivariate Cox regression.

Category	Cancer specific survival		Overall survival	
	HR (95% CI)	*P-*value	HR (95% CI)	*P-*value
Age (year)	1.12 (1.09-1.14)	<0.001	1.10 (1.09-1.10)	<0.001
Diagnosis year	0.83 (0.74-0.92)	0.001	0.95 (0.92-0.98)	0.002
Sex				
Female	Ref	0.889	Ref	<0.001
Male	1.04 (0.57-1.91)		1.68 (1.41-2.00)	
Race				
White	Ref		Ref	
Black	0.96 (0.23-4.00)	0.954	1.99 (1.54-2.56)	<0.001
Other	0.87 (0.35-2.18)	0.762	0.71 (0.51-0.98)	0.038
Multifocality				
No	Ref	0.332	Ref	0.351
Yes	0.75 (0.42-1.34)		0.92 (0.77-1.10)	
Extrathyroidal extension				
No	Ref		Ref	
Minimal extension	1.71 (0.58-5.07)	0.334	0.98 (0.56-1.71)	0.930
Gross extension	2.60 (1.22-5.56)	0.014	1.67 (1.15-2.41)	0.006
CLNM				
No	Ref		Ref	
N1a	4.69 (1.92-11.5)	0.001	1.29 (0.85-1.94)	0.228
N1b	10.11 (4.64-22.03)	<0.001	2.40 (1.68-3.43)	<0.001
N1 (NOS)	2.54 (0.55-11.70)	0.231	2.17 (1.22-3.87)	0.009
Distant metastasis				
No	Ref	<0.001	Ref	<0.001
Yes	24.54 (11.17-53.89)		6.19 (3.54-10.82)	
Histotype				
PTC	Ref	0.736	Ref	0.127
FVPTC	0.90 (0.49-1.67)		1.14 (0.96-1.35)	
RAI				
No	Ref	0.011	Ref	0.116
Yes	2.56 (1.25-5.25)		0.83 (0.67-1.04)	
Chemotherapy				
No	Ref	0.001	Ref	0.031
Yes	14.10 (3.03-65.70)		3.64 (1.12-11.80)	
Surgery				
TL	Ref	0.095	Ref	0.116
TT	0.53 (0.25-1.12)		0.86 (0.72-1.04)	

PTMC, papillary thyroid microcarcinoma; HR (95% CI), hazard ratio (95% confidence interval); CLNM, cervical lymph node metastasis; PTC, papillary thyroid carcinoma; FVPTC, follicular variant papillary thyroid carcinoma; TL, thyroid lobectomy; TT, total thyroidectomy; RAI, radioactive iodine.

In the univariate Cox regression analysis, TT was associated with improved OS compare with TL with HR (95% CI) of 0.74 (0.62-0.88) (**Supplementary Table 1**). In the multivariate Cox regression model, there was a trend toward a better prognosis in CSS and OS of TT over TL with HR (95% CI) of 0.53 (0.25-1.12) and 0.86 (0.72-1.04), respectively. However, the differences were not statistically different (**Table 2**).

### Kaplan–Meier Curves Before and After PSM

**Figure 1:**
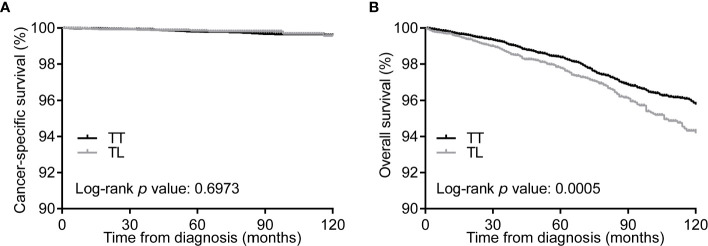
Kaplan-Meier curves of CSS **(A)** and OS **(B)** of PTMC patients undergoing TT *versus* TL before propensity score matching. CSS, cancer specific survival; OS, overall survival; PTMC, papillary thyroid microcarcinoma; TT, total thyroidectomy; TL, thyroid lobectomy.

**Figure 2:**
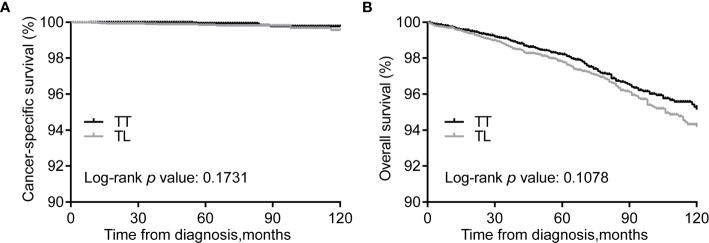
Kaplan-Meier curves of CSS **(A)** and OS **(B)** of PTMC patients undergoing TT *versus* TL after propensity score matching. CSS, cancer specific survival; OS, overall survival; PTMC, papillary thyroid microcarcinoma; TT, total thyroidectomy; TL, thyroid lobectomy.

Kaplan–Meier curves showed no differences in CSS between the TT and TL groups (**Figure 1A**). However, the median OS of TT was significantly longer than that of TL before matching (**Figure 1B**). After balancing the baseline characteristics between TL and TT, the differences between the two groups were significantly reduced (**Supplementary Figure 2**). The matched process yielded a total of 6929 paired cases. The differences in baseline covariates were well balanced after matching (**Supplementary Table 2**). However, there were no significant differences in CSS and OS between patients with TT and TL (**Figures 2A, B**).

**Figure 3:**
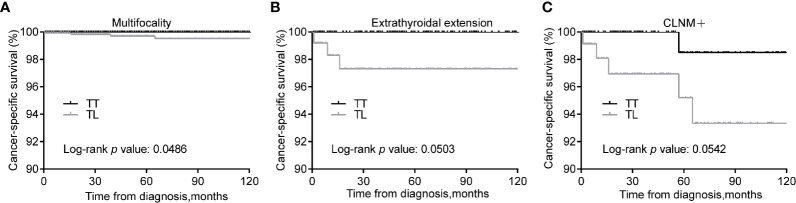
Kaplan-Meier curves of CSS of PTMC underwent TT *versus* TL among patients with multifocality **(A)**, extrathyroidal extension **(B)**, and CLNM **(C)** after propensity score matching. CSS, cancer specific survival; PTMC, papillary thyroid microcarcinoma; TT, total thyroidectomy; TL, thyroid lobectomy; CLNM, cervical lymph node metastasis.

We expected that patients with risk clinicopathologic characteristics may benefit from TT. Patients with tumors of multifocality can gain improved CSS from TT over TL (*p* = 0.049) after matching (**Figure 3A**). In addition, patients with tumors of extrathyroidal extension and CLNM showed marginally improved CSS from TT over TL (*P* = 0.050 and 0.054, respectively) (**Figures 3B, C**).

### Subgroup Analysis by Multivariate Cox Regression Analysis

In consideration of the tread toward improved prognosis from TT, we further performed subgroup analysis to identify those who might benefit from TT over TL. Compared with TL, TT was associated with improved CSS for patients < 55 years, those with tumors of gross extrathyroidal extension, N1b, and those not receiving RAI with HR (95% CI) of 0.13 (0.02-0.81), 0.12 (0.02-0.66), 0.11 (0.02-0.64), 0.34 (0.13-0.90), respectively. In addition, TT was associated with marginally improved OS for patients with N1b,distant metastasis, and non-RAI with HR (95% CI) of 0.30 (0.08-1.08) and 0.06 (0.001-2.94), and 0.84 (0.70-1.02), respectively (**Table 3**).

**Table 3:** The subgroup analysis of the prognosis of PTMC patients treated with TT *versus* TL by multivariate Cox regression.

Category	Cancer specific survival		Overall survival	
	HR (95% CI)	*P-*value	HR (95% CI)	*P-*value
Age < 55 years				
TL	Ref	0.028	Ref	0.327
TT	0.13 (0.02-0.81)		0.82 (0.56-1.22)	
Gross extrathyroidal extension				
TL	Ref	0.014	Ref	0.106
TT	0.12 (0.02-0.66)		0.42 (0.15-1.20)	
CLNM (N1b)				
TL	Ref	0.014	Ref	0.065
TT	0.11 (0.02-0.64)		0.30 (0.08-1.08)	
Distant metastasis				
TL	Ref	0.297	Ref	0.061
TT	0.12 (0.002-6.22)		0.06 (0.001-2.94)	
Non-RAI				
TL	Ref	0.030	Ref	0.077
TT	0.34 (0.13-0.90)		0.84 (0.70-1.02)	

The multivariate Cox regression was adjusted for all the other covariates.

PTMC, papillary thyroid microcarcinoma; TT, total thyroidectomy; TL, thyroid lobectomy; HR (95% CI), hazard ratio (95% confidence interval); CLNM, cervical lymph node metastasis; RAI, radiotherapy.

### The Prognosis of PTMC With Two or More Risk Clinicopathologic Factors

As shown above, PTMC patients with one risk factor gained survival benefit from TT. It’s interesting to investigate the prognosis of patients with multiple risk factors. In the multivariate Cox regression model, TT can significantly improve CSS of patients with tumors of multifocality & gross extrathyroidal extension, multifocality & N1b, multifocality & extrathyroidal extension & CLNM, extrathyroidal extension & CLNM, or CLNM & age < 55 years compared with TL with HR (95% CI) of 0.13 (0.02-0.82), 0.04 (0.004-0.32), 0.08 (0.01-0.69), 0.13 (0.02-0.73), and 0.10 (0.01-0.82), respectively. Of patients with tumors of multifocality & N1b, TT was associated with improved OS compared with TL with HR (95% CI) of 0.18 (0.04-0.96) (**Table 4**).

**Table 4:** The prognosis of PTMC patients according to the extent of surgery by subgroup analysis stratified by clinicopathologic factors by multivariate Cox regression.

Category	Cancer specific survival			Overall survival	
	HR (95% CI)		*P-*value	HR (95% CI)	*P-*value
Multifocality & gross extrathyroidal extension					
TL (*n* = 30)	Ref		0.030	Ref	0.136
TT (*n* = 526)	0.13 (0.02-0.82)			0.36 (0.10-1.37)	
Multifocality & CLNM (N1b)					
TL (*n* = 13)	Ref		0.003	Ref	0.045
TT (*n* = 754)	0.04 (0.004-0.32)			0.18 (0.04-0.96)	
Multifocality & extrathyroidal extension & CLNM					
TL (*n* = 8)	Ref		0.022	Ref	0.100
TT (*n* = 521)	0.08 (0.01-0.69)			0.22 (0.04-1.34)	
Extrathyroidal extension & CLNM					
TL (*n* = 21)	Ref		0.021	Ref	0.061
TT (*n* = 734)	0.13 (0.02-0.73)			0.26 (0.06-1.06)	
Age < 55 years & CLNM					
TL (*n* = 88)	Ref		<0.001	Ref	0.189
TT (*n* = 2675)	0.10 (0.01-0.82)			0.39 (0.09-1.60)	

The multivariate Cox regression was adjusted for all the other covariates in each category.

PTMC, papillary thyroid microcarcinoma; TT, total thyroidectomy; TL, thyroid lobectomy; HR (95% CI), hazard ratio (95% confidence interval); CLNM, cervical lymph node metastasis.

**Figure 4:**
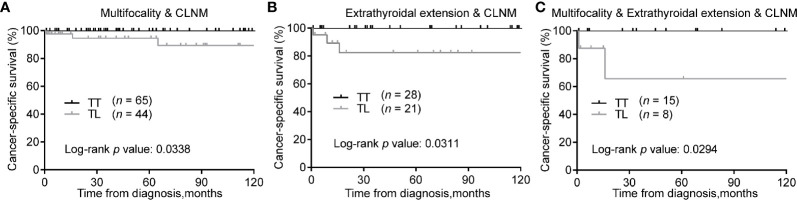
Kaplan-Meier curves of CSS of PTMC underwent TT *versus* TL among patients with multifocality & CLNM **(A)**, extrathyroidal extension & CLNM **(B)**, and multifocality & extrathyroidal extension & CLNM **(C)** after propensity score matching. CSS, cancer specific survival; PTMC, papillary thyroid microcarcinoma; TT, total thyroidectomy; TL, thyroid lobectomy; CLNM, cervical lymph node metastasis.

Consistently, in the matched cohort, TT was associated with improved CSS of patients with tumors of multifocality & CLNM (**Figure 4A**), extrathyroidal extension & CLNM (**Figure 4B**), or multifocality & extrathyroidal extension & CLNM (**Figure 4C**) (Log-rank *P* < 0.05 for all).

## Discussion

In the present study, we investigated the extent of surgery and the prognosis of patient with unilateral PTMC. TT was not associated with improved CSS and OS compared with TL in the total population after PSM. However, TT predicted better CSS for patients < 55 years, those with tumors of gross extrathyroidal extension, N1b, and not receiving RAI. After balancing the covariates between the TL and TT groups, we found that TT can improve CSS for patients with tumors of multifocality, extrathyroidal extension, and CLNM compared with TL. Importantly, we found that patient with multiple risk clinicopathologic factors like CLNM, extrathyroidal extension, and multifocality were more likely to benefit from TT over TL.

The optimal extent of surgery for PTMC has been controversial. Most of the single institutional studies and meta-analysis failed to discern any differences in prognosis of patients with PTMC underwent TT or TL, which might result from the indolent behavior of PTMC, the short-term follow-up duration, and the relatively small sample size (13–15). There may be a trend toward lower mortality rate of TT than TL. However, the limited number of mortality events prevented establishing a definitive correlation between the extent of surgery and prognosis of patients with PTMC (14).

Previous studies found that the recurrence and survival were not statistically different between PTMC patients undergoing TT and TL by the National Cancer Data Base (1985–1998) (16). However, some variables such as multifocality, extrathyroidal extension, pathological type, chemotherapy and RAI were missing or incomplete, and subgroup analysis were not performed. Lee et al. did not find any significant differences in the risk of death and locoregional recurrence between TT and TL in a matched cohort with 506 paired PTMC patients from 1986 to 2006 (15). Some single institutional studies found that the recurrence rate of patients undergoing TT was similar with those undergoing TL (3, 15). However, a recent meta-analysis showed that TT was associated with lower recurrence rates than TL (14, 17). For PTMC of multifocality, TL may result in a higher rate of thyroid bed and lymph node recurrence than TT (18). The low recurrence rate of TT might result from a more radical resection of the contralateral thyroid lobe and cervical lymph nodes (5, 19), while transient and permanent hypoparathyroidism was higher for TT than TL (5).

We found that patients undergoing TT were more likely to be younger, and present with tumors of multifocality, extrathyroidal extension, CLNM, and distant metastasis. These features were associated with nodal metastasis, tumor recurrence, and unfavorable prognosis of patients (7, 8, 20). We found that patients undergoing TT showed a trend toward improved CSS and OS compared with patients receiving TL. Relative treatment effects may vary according to the heterogeneous study population, certain high-risk subsets may benefit most from the treatment (21). We thus expected that a subpopulation of PTMC patients may benefit from TT.

The subgroup analysis revealed that patients < 55 years, those with tumors of gross extrathyroidal extension had improved CSS from TT compared with TL. Younger age and extrathyroidal extension were risk factors for CLNM (7, 8). Of note, TT failed to improve the prognoses of patients with minimal extrathyroidal extension and N1a. For patients with N1b, TT significantly improved CSS of patients compared with TL. These findings highlighted the importance of detecting nodal metastasis in the lateral neck. The preferred hierarchy of treatment for PTC with distant metastasis includes TT, nodal dissection, postoperative RAI therapy, and thyrotropin inhibition therapy. As for refractory disease, kinase inhibitors were recommended (4). We found that patients with distant metastasis may benefit from TT over TL. However, the number of patients with distant metastasis was relatively small and the result needs to be validated in the following studies. The present study found that patients not receiving postoperative RAI might benefit from TT with improved CSS compared with TL. TT might facilitate to eradicate disease recurrence of the contralateral lobe, and potential metastatic lymph nodes, which was beneficial for those not receiving RAI.

We did not observe any differences in the prognosis between TT and TL for PTMC patient with multifocality in the multivariate model, which was consistent with a previous study (22). However, after PSM between TL and TT, TT showed improved CSS for multifocal tumor in unilateral PTMC patients compared with TL, while the OS was similar. TL may be a safe treatment approach for selected unilateral PTMC patients with multifocal and node-negative tumors (3), which was consistent with our results. The prognostic significance of multifocal tumors in PTC remains controversial (23). However, when tumors of multifocality together with CLNM, extrathyroidal extension, or both were presented, TT was associated with improved CSS compared with TL. Therefore, a more radical surgical treatment may be considered for tumors with more risk factors.

The study should be interpreted in consideration of several limitations. First, selection bias was inevitable even though we adjusted the covariates and performed PSM analysis. In addition, data like disease recurrence and thyroid-stimulating hormone inhibition were not available in the database. Additionally, the results of subgroup analysis should be interpreted with caution for the limited samples and events evaluated. Last but not least, the occult thyroid cancer in the contralateral thyroid lobe and the exact number of metastatic lymph nodes may also influence the outcome. The strengths of this study lie in the latest and largest samples to date, comprehensive variables adjusted, and subgroup analysis together with PSM analysis.

In conclusion, we for the first time investigated the association between the extent of thyroid surgery and prognosis of unilateral PTMC patients. The present results suggested that there was no statistical difference in prognosis between TL and TT for unilateral PTMC patients. TL is appropriate for unilateral PTMC without risk factors. However, PTMC patients with risk features such as a younger age, multifocality, gross extrathyroidal extension, and N1b may benefit from TT over TL, especially for those with multiple risk factors. These findings may have an impact on the treatment of unilateral PTMC. Large sample size and long-term follow-up studies are warranted to validate the present findings.

## Data Availability Statement

The original contributions presented in the study are included in the article/**Supplementary Material**/Further inquiries can be directed to the corresponding author.

## Author Contributions

HZ: conception, data acquisition. HZ and LC: data analysis and drafting the article. HZ and LC: revised it critically for important intellectual content. HZ: investigation, project administration, and supervision. All authors contributed to the article and approved the submitted version.

## Funding

This work was funded by the Fundamental Research Funds for the Central Universities (grant number 2042020kf0063).

## Conflict of Interest

The authors declare that the research was conducted in the absence of any commercial or financial relationships that could be construed as a potential conflict of interest.
