# Supplementary material for: Extent of Surgery and the Prognosis of Unilateral Papillary Thyroid Microcarcinoma
Source: Front Endocrinol (Lausanne). 2021 Jun 16;12:655608. doi: 10.3389/fendo.2021.655608 (PMC8242954; doi:10.3389/fendo.2021.655608)
Supplement: Supplementary file 1 [file DataSheet_1.docx]

Supplementary Material


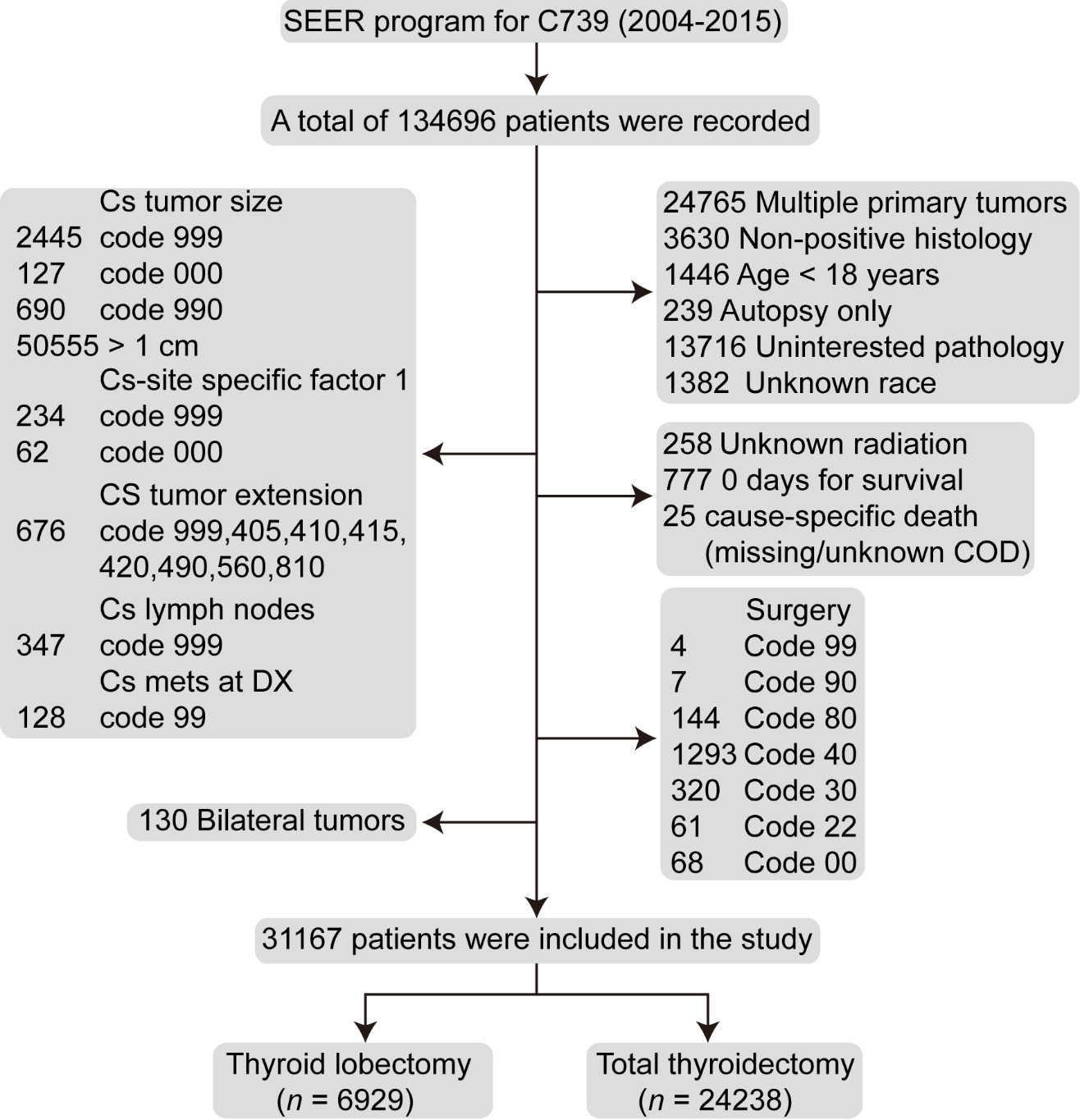


**Supplementary Figure 1.** The flowchart of the selection process.


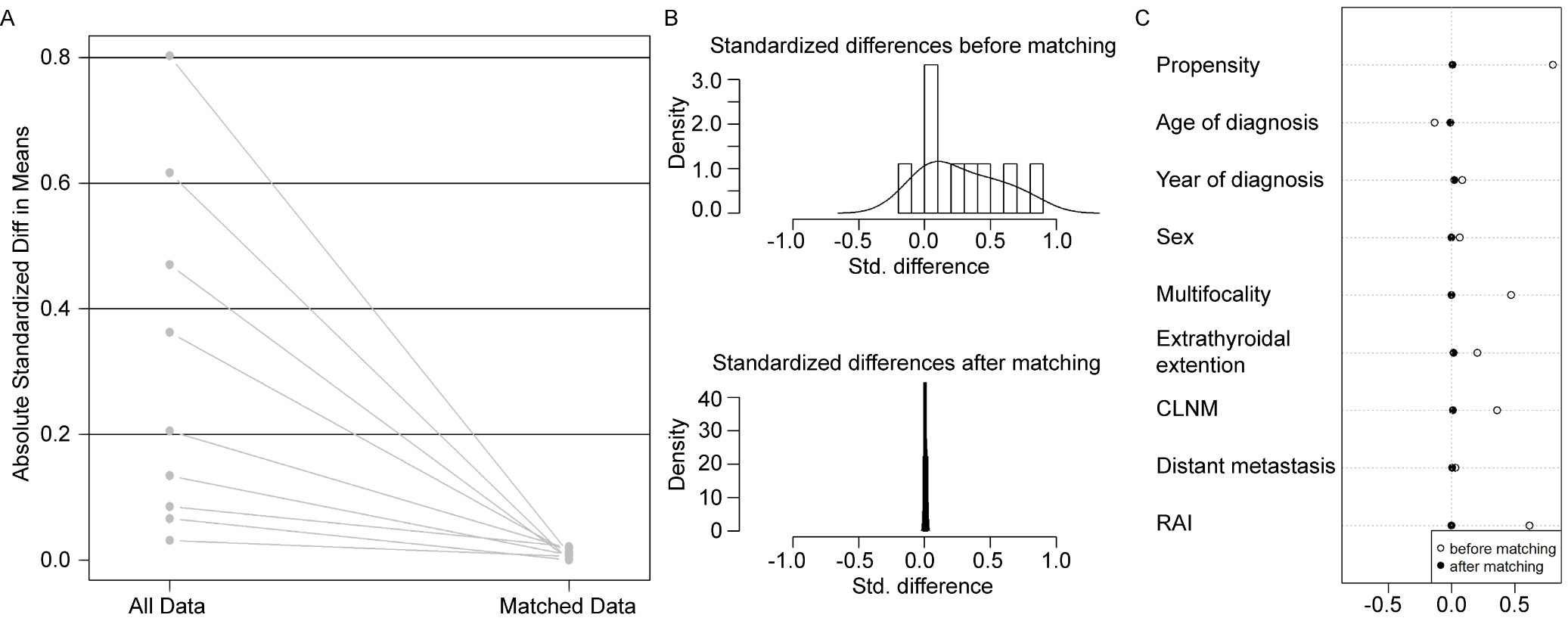


**Fig. 2** Standardized differences of baseline variables of PTMC patients undergoing TL and TT after propensity score matching. (A) The line graph shows standardized differences after matching. (B-C) The histogram and scatter gram show the absolute standardized differences after matching. PTMC, papillary thyroid microcarcinoma; TT, total thyroidectomy; TL, thyroid lobectomy; CLNM, cervical lymph node metastasis; RAI, radioactive iodine.

| **Table 1** The predictors for cancer specific survival and overall survival of PTMC patients in a univariate Cox proportional hazards regression model | | | | |
| --- | --- | --- | --- | --- |
| Category | Cancer specific survival | | Overall survival | |
|  | HR (95% CI) | *P-*value | HR (95% CI) | *P-*value |
| Age (year) | 1.11 (1.08-1.13) | < 0.001 | 1.10 (1.09-1.11) | < 0.001 |
| Diagnosis year | 0.85 (0.76-0.94) | 0.003 | 0.97 (0.93-1.00) | 0.054 |
| Sex |  |  |  |  |
| Female | Ref | < 0.001 | Ref | < 0.001 |
| Male | 2.89 (1.68-4.94) |  | 2.17 (1.83-2.58) |  |
| Race |  |  |  |  |
| White | Ref |  | Ref |  |
| Black | 0.57 (0.14-2.32) | 0.429 | 1.84 (1.43-2.38) | < 0.001 |
| Other | 1.11 (0.48-2.60) | 0.806 | 0.71 (0.51-0.98) | 0.035 |
| Multifocality |  |  |  |  |
| No | Ref | 0.293 | Ref | 0.037 |
| Yes | 1.33 (0.78-2.24) |  | 0.84 (0.71-0.99) |  |
| Capsular extension | |  |  |  |
| No | Ref | < 0.001 | Ref | 0.073 |
| Yes | 6.30 (3.53-11.22) |  | 1.31 (0.98-1.77) |  |
| CLNM |  |  |  |  |
| No | Ref | < 0.001 | Ref | 0.150 |
| Yes | 7.67 (4.56-12.90) |  | 1.19 (0.94-1.50) |  |
| Distant metastasis | |  |  |  |
| No | Ref | < 0.001 | Ref | < 0.001 |
| Yes | 117.77 (59.48-233.19) |  | 12.91 (7.60-21.93) |  |
| Histotype |  |  |  |  |
| PTC | Ref | 0.788 | Ref | 0.004 |
| FVPTC | 0.92 (0.51-1.66) |  | 1.28 (1.08-1.51) |  |
| Radiation |  |  |  |  |
| No | Ref | < 0.001 | Ref | < 0.001 |
| Yes | 3.75 (2.19-6.44) |  | 0.71 (0.59-0.85) |  |
| Chemotherapy |  |  |  |  |
| No | Ref | < 0.001 | Ref | 0.004 |
| Yes | 40.50 (9.87-166.17) |  | 5.26 (1.69-16.36) |  |
| Surgery |  |  |  |  |
| TL | Ref | 0.698 | Ref | 0.001 |
| TT | 1.14 (0.60-2.14) |  | 0.74 (0.62-0.88) |  |
| PTMC, papillary thyroid microcarcinoma; HR (95% CI), hazard ratio (95% confidence interval); CLNM, cervical lymph node metastasis; PTC, papillary thyroid carcinoma; FVPTC, follicular variant papillary thyroid carcinoma; TL, thyroid lobectomy; TT, total thyroidectomy | | | | |

| **Table 2** The clinicopathologic characteristics of patients with PTMC treated with TL versus TT after propensity score matching (*n* = 6929 pairs) | | | |
| --- | --- | --- | --- |
| Category | TL | TT | *P-*value |
| Age (year) | 51 (41-61) | 51 (41-60) | 0.750 |
| < 55 years | 4179 (60.3) | 4178 (60.3) | 0.986 |
| ≥ 55 years | 2750 (39.7) | 2751 (39.7) |  |
| Year of diagnosis | 2011 (2007-2013) | 2011 (2008-2013) | 0.281 |
| Sex |  |  |  |
| Male | 1322 (19.1) | 1322 (19.1) | 1.000 |
| Female | 5607 (80.9) | 5607 (80.9) |  |
| Race |  |  |  |
| White | 5753 (83.0) | 5792 (83.6) | < 0.001 |
| Black | 463 (6.7) | 554 (8.0) |  |
| Other | 713 (10.3) | 583 (8.4) |  |
| Multifocality |  |  |  |
| No | 5705 (82.3) | 5702 (82.3) | 0.947 |
| Yes | 1224 (17.7) | 1227 (17.7) |  |
| Capsule extension |  |  |  |
| No | 6805 (98.2) | 6773 (97.7) | 0.053 |
| Yes | 124 (1.8) | 156 (2.3) |  |
| CLNM |  |  |  |
| No | 6814 (98.3) | 6785 (97.9) | 0.069 |
| Yes | 115 (1.7) | 144 (2.1) |  |
| Distant metastasis |  |  |  |
| No | 6923 (99.9) | 6921 (99.9) | 0.593 |
| Yes | 6 (0.1) | 8 (0.1) |  |
| Histotype |  |  |  |
| PTC | 4842 (69.9) | 4969 (71.7) | 0.018 |
| FVPTC | 2087 (30.1) | 1960 (28.3) |  |
| Radiation |  |  |  |
| No | 6582 (95.0) | 6582 (95.0) | 1.000 |
| Yes | 347 (5.0) | 347 (5.0) |  |
| Chemotherapy |  |  |  |
| No | 6925 (99.9) | 6919 (99.9) | 0.109 |
| Yes | 4 (0.1) | 10 (0.1) |  |
| PTMC, papillary thyroid microcarcinoma; TL, thyroid lobectomy; TT, total thyroidectomy; CLNM, cervical lymph node metastasis; FVPTC, follicular variant papillary thyroid carcinoma; PTC, papillary thyroid carcinoma | | | |
